# Heat treatment combined with hybrid exercises retraining mitigates cellular markers of protein turnover after hindlimb suspension in male mice: A pilot study

**DOI:** 10.1113/EP093111

**Published:** 2026-06-16

**Authors:** Tom Normand‐Gravier, Robert Solsona, Christelle Bertrand‐Gaday, Margot Issertine, Florence Sabatier, Flavie Arnould, Sébastien Racinais, Fabio Borrani, Henri Bernardi, Anthony M. J. Sanchez

**Affiliations:** ^1^ Faculty of Sports Sciences, Laboratoire Interdisciplinaire Performance Santé Environnement de Montagne (LIPSEM) University of Perpignan Via Domitia (UPVD), UR4640 Font‐Romeu France; ^2^ Dynamique du Muscle et Métabolisme (DMEM) University of Montpellier, INRAE, UMR866 Montpellier France; ^3^ Institute of Sport Sciences, Biomechanics & Integrative Physiology of Exercise Dynamics (BIPED) laboratory University of Lausanne Lausanne Switzerland; ^4^ Racinais is Environmental Stress Unit CREPS Montpellier—Font‐Romeu Montpellier France

**Keywords:** atrophy, autophagy, exercise, heat therapy, mTOR, skeletal muscle, thermal intervention

## Abstract

Recent evidence suggests that heat treatment (HT) and resistance training can limit skeletal muscle mass loss during immobilization. However, the effects of repeated HT sessions combined with hybrid exercises (EX), which promote both endurance and resistance responses, on muscle protein turnover following hindlimb unloading (HU) remain unexplored. This study investigated the effects of reloading strategies using HT and/or EX on protein synthesis, ribosome biogenesis and autophagic markers. Eight‐week‐old male C57BL/6J mice underwent HU (3 weeks), HU+HT (1 week, 5 sessions, 30 min, 40°C), HU+EX (5 sessions of high‐intensity inclined treadmill running), HU+EX+HT or HU+REL (passive reloading). HU+HT did not induce additional effects compared with the HU+REL group concerning protein synthesis rates, although one marker of protein synthesis (phosphorylated ribosomal protein S6) tended to increase. HU+HT did not lead to an increase in rRNA content. HU+EX induced a concomitant decrease in the mitochondrial fission markers phosphorylated dynamin‐related protein 1 and phosphorylated AMP‐activated protein kinase, suggesting improved mitochondrial efficiency. However, no additional effects were observed for rRNA or heat shock proteins compared with the HU+REL group. Finally, exercise followed by heat treatment post‐HU (HU+EX+HT) appears to be detrimental, as protein synthesis rates did not increase, despite an increase in rRNA content. Thus, at this sampling time point (14 days post‐HU), neither heat therapy nor hybrid retraining produced additional effects on the studied markers compared with a normal resumption of activity. In contrast, the sequential application of the two stressors may inhibit post‐HU muscle mass recovery, as evidenced by the absence of increased protein synthesis rates.

## Abstract (highlights)

- **What is the central question of this study?**
  What are the effects of reloading strategies (hybrid exercises with/without heat treatment) on protein synthesis, ribosome biogenesis and autophagic markers after hindlimb unloading?
- **What is the main finding and its importance?**
  After 3 weeks, hindlimb unloading drastically decreased global protein synthesis and ribosomal DNA transcription. Heat therapy and hybrid retraining induced only a few additional effects compared to normal reloading when applied individually. Exercise followed by heat appeared to inhibit the increase in protein synthesis induced by heat therapy or passive reloading.

## 1 INTRODUCTION

Skeletal muscle accounts for approximately 40% of total body weight (Janssen et al., 2000) and ensures movement and posture (Kim et al., 2016). Beyond its functional role, skeletal muscle is a critical organ for whole body health, and its dysfunction can trigger different pathologies, such as type 2 diabetes and fatty liver (Al‐Ozairi et al., 2021; Kim & Kim, 2020). Hindlimb unloading (HU), an experimental protocol in rodents that mimics human skeletal immobilization, has deleterious effects on muscle homeostasis by decreasing muscle mass, fibre cross‐sectional area (CSA) and strength (Haida et al., 1989; Oliveira et al., 2019).

Fourteen days of HU leads to unbalanced protein turnover, decreased protein synthesis and increased protein degradation, favouring skeletal muscle atrophy (Baehr et al., 2016, 2017). Changes in mitochondrial dynamics (i.e., mitochondrial fusion and fission) also contribute to aggravating skeletal muscle atrophy during immobilization (Hafen et al., 2019). Dynamin‐related protein 1 (DRP1), a mitochondrial fission marker, contributes to the removal of damaged mitochondria by separating them into different organelles (Dulac et al., 2020). It has been previously reported that mitochondrial fission is promoted by the phosphorylation of DRP1 at Ser616 (Taguchi et al., 2007) by AMP‐activated protein kinase (AMPK), altering mitochondrial function (Dulac et al., 2021). The autophagy pathway removes non‐functional cellular components, such as organelles (e.g., ribosomes and mitochondria) and lipid droplets (Sanchez et al., 2015). Endurance exercise is well recognized for enhancing autophagic flux (i.e., p62 content and LC3‐B II/I ratio) to promote adequate cell component turnover (Sanchez et al., 2014). Importantly, autophagy, especially mitophagy (i.e., autophagy of mitochondria), during immobilization appears to be required to limit muscle mass loss due to defective mitochondria accumulation and the subsequent enhanced oxidative stress (Rahman et al., 2025).

Resistance training promotes strength improvements and muscle hypertrophy (Lopez et al., 2021) through the activation of the mechanistic target of the rapamycin complex 1 (mTORC1) pathway, leading to an increase in protein synthesis (Morita et al., 2015). In this context, exercise has been proposed as a reloading strategy to restore muscle mass following HU (Mirzoev, 2020). Resistance training and voluntary wheel‐running seem to be promising reloading strategies to favour muscle recovery following HU, by increasing muscle mass, CSA, total rRNA content and mitochondrial function (Figueiredo et al., 2021; Hanson et al., 2010; Ishihara et al., 2004). As endurance and resistance training promote different but complementary cellular responses, combining endurance and resistance exercise can lead to cross‐responses within the muscle cell, characterized by muscle hypertrophy due to elevated protein synthesis and improved oxidative capacity resulting from enhanced mitochondrial biogenesis. On the other hand, recent evidence has shown that heat treatment (HT), also called ‘kaumatherapy’, such as passive muscle heating, hot‐water immersion (HWI) or pulsed shortwave diathermy (Normand‐Gravier, Solsona, Dablainville et al., 2025), can act as an exercise mimetic for muscle strength and hypertrophy in sedentary populations (Rodrigues et al., 2020) and can limit muscle mass loss during immobilization (Hafen et al., 2019; Labidi et al., 2024). In this regard, it has been demonstrated that acute heat stress increased the activity of the mTORC1 pathway, with enhanced phosphorylation of mTOR, ribosomal protein S6 (RPS6) and the eukaryotic translation initiation factor 4E‐binding protein 1 (4E‐BP1) following passive muscle heating (Ihsan et al., 2020; Kakigi et al., 2011). Heat shock proteins (HSPs) are chaperone proteins that can limit the aggregation or denaturation of other proteins and preserve cellular function (Dahiya & Buchner, 2019). These proteins can be activated by different stressors, such as heat stress or exercise (Archer et al., 2018; Hafen et al., 2019) and can also stimulate protein synthesis (Craig et al., 1993). Hence, modulation of HSP expression can impact protein synthesis. Interestingly, modulation of muscle hypertrophy markers (mTOR, HSP70) and muscle inflammation markers (e.g., nuclear factor‐κB (NF‐κB)) as well as effects on maximal isometric strength, muscle mass and CSA have been observed with regular HT performed during or after HU, an immobilization period or in the context of disuse‐muscle atrophy or muscle damage (AlSabagh et al., 2022; Dablainville et al., 2025; Goto et al., 2004; Hafen et al., 2019; Labidi et al., 2024; Naito et al., 2000; Nonaka et al., 2015; Selsby & Dodd, 2005). More specifically, after an immobilization of 7 days, HT (30 min, 41–41.5°C on alternating days) performed during 7 days of reloading led to a combined increase of HSP expression and soleus muscle mass and to an attenuation of oxidative damage in rat soleus muscle (Selsby et al., 2007), indicating that HT is an effective intervention to promote muscle mass recovery following immobilization. This protocol raised the core temperature by ∼1–2°C above the normal 36.5–38°C, sufficient to trigger systemic HSP responses without overt stress, highlighting the importance of core, rather than local, heating for promoting recovery mechanisms (Eng et al., 2014). Compared with humans, mice have a high surface‐area‐to‐volume ratio, which permits a rapid increase in core temperature in response to environmental heat stress and makes the thermoregulatory context relevant when interpreting heat shock responses.

Ribosomes, composed of ribosomal RNAs (rRNAs) and proteins, play a fundamental role in muscle cells by translating mRNA into proteins (Figueiredo & McCarthy, 2019). The ribosome is composed of two subunits (40S and 60S), each containing different rRNAs and ribosomal proteins (Figueiredo & McCarthy, 2019). In mice, 28S rRNA represents the major component of the large subunit (60S), whereas the small subunit (40S) has 18S rRNA (Figueiredo & McCarthy, 2019). It has been shown that an increase in ribosomal content leads to increased translational capacity (Figueiredo & McCarthy, 2019), thereby increasing protein synthesis and favouring muscle hypertrophy (Chaillou, 2019; Solsona & Sanchez, 2020). Notably, the upstream binding factor 1 (UBF‐1) protein, encoded by the upstream binding transcription factor (*UBTF*) gene, is a transcription factor involved in the regulation of rDNA transcription. This protein is crucial for the ribosome production process but its role during atrophy remains unknown.

Thus, the combination of HT and hybrid retraining exercises that aim to promote both endurance and resistance responses (EX), should be a promising strategy to positively regulate protein synthesis after HU. Hence, this study aimed to identify the associations between HU, HU+REL, HU+HT, HU+EX and HU+EX+HT on rRNA, protein turnover, ribosome biogenesis and mitochondrial fission. We postulated that (i) HU would decrease protein synthesis flux and rRNA content, (ii) HT and EX post‐HU would have significant effects on protein turnover and ribosome biogenesis markers, and (iii) the sequential application of both stressors would result in an increased response compared with each stressor applied individually.

## 2 METHODS

### 2.1 Ethical approval

All animal experiments were performed in accordance with European directives and approved by the Ethical Committee of Region Languedoc‐Roussillon no. CEEA‐36 and the French Ministry of Higher Education and Research (no. 43054_2023042015168782). Experiments were conducted according to the guidelines laid down by the institution's animal welfare committee and comply with *Experimental Physiology*’s policies regarding animal experiments.

### 2.2 Design

**FIGURE 1:**
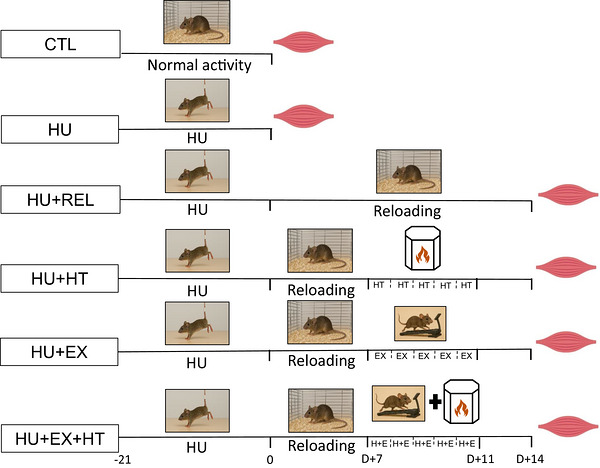
Experimental design. The HU group underwent hindlimb unloading for 3 weeks, and gastrocnemius muscles were dissected immediately after the 3‐week unloading period (D0), as was done for the CTL group that remained in cage with normal activity during the HU period (D0). In HU+REL, HU+HT, HU+EX and HU+EX+HT, mice underwent HU for 3 weeks (D0) followed by a 2‐week recovery period (D+14 post‐HU). Gastrocnemius muscles were dissected after a normal reloading of 2 weeks in HU+REL (D+14 post‐HU). In this same period, the remaining groups received one of the following interventions after 1 week of normal reloading (D+7): 5 sessions of high‐intensity inclined treadmill running (HU+EX), heat treatment (HU+HT) or sequential application of both stressors (HU+EX+HT). Gastrocnemius muscles were dissected 3 days after the completion of the last EX or HT session for HU+HT, HU+EX and HU+EX+HT groups (D+14 post‐HU).

Forty‐six 8‐week‐old male C57BL/6J mice (Janvier Labs, Saint‐Berthevin, France) were used in this study. Given the fact that young female mice exhibit higher baseline levels of autophagy and an attenuated autophagic clearance following exercise compared to age‐matched males (Triolo et al., 2022), we exclusively used male mice to avoid inter‐sex variability in autophagic responses. Mice were individually housed in cages under a controlled 12:12‐h light–dark cycle, with the dark phase occurring between 10.00 h and 22.00 h, in a temperature‐regulated environment (22 ± 1°C). They had unrestricted access to water and were fed a standardized diet (A04, SAFE, Augy, France). Following a 2‐week acclimatization period to the housing conditions and light–dark cycle, animals were randomly distributed into six experimental groups: (i) CTL (control group, *n* = 8), (ii) HU (*n* = 8), (iii) HU+REL (reloading) (*n* = 6), (iv) HU+HT (*n* = 8), (v) HU+EX (*n* = 8), and (vi) HU+EX+HT (*n* = 8). Mice were euthanized by cervical dislocation without anaesthesia according to the following timing: after 3 weeks of normal activity for CTL (D0), immediately after the HU period of 3 weeks for HU group (D0), after 3 weeks of HU and normal reloading of 2 weeks for the HU+REL group (D+14). For HU+EX, HU+HT and HU+EX+HT groups, mice were euthanized after 3 weeks of HU, 1 week of normal reloading and 1 week of HT or EX (daily HT or EX for 5 days). To avoid the acute effects of the last HT or EX session, mice were euthanized 3 days after the completion of the last HT or EX session (D+14). Durations of 2–3 weeks of HU are widely used in adult mice to induce a robust yet reversible atrophic phenotype in skeletal muscles, with marked mitochondrial and fibre‐type remodelling and preserved capacity for subsequent recovery (Ferreira et al., 2011). Early recovery after unloading is characterized by rapid changes in muscle mass, damage markers, proteolytic activity and mitochondrial content within the first 7 days of reloading, indicating that 1 week is sufficient to capture pronounced adaptive responses while the muscle remains in an early recovery state (Roberson et al., 2020). The experimental design is detailed in Figure 1. Gastrocnemius muscles were harvested, as this muscle is a major hindlimb locomotor muscle with a mixed but predominantly fast‐twitch phenotype, which is highly sensitive to unloading, immobilization and subsequent reloading or therapeutic interventions (Chacon‐Cabrera et al., 2016). Muscles were snap‐frozen in liquid nitrogen and stored at −80°C until analysis.

Core body temperature was assessed by manually placing mice on a clean cage lid. The tail was gently elevated to expose the rectal area. A rectal temperature probe (RET‐3 model, Physitemp, Clifton, NJ, USA), lubricated with petroleum jelly, was carefully inserted 20 mm into the rectum, measured from the tail's base region. The positioning process required around 15 s to minimize stress. Following insertion, the probe was held in place for 5 s to allow for temperature stabilization before recording. Rectal temperature was measured immediately after the completion of HT, EX or EX+HT session.

### 2.3 HU

For three weeks, mice in HU, HU+REL, HU+HT, HU+EX and HU+EX+HT groups were bilaterally hindlimb unloaded, as previously described (Marzuca‐Nassr et al., 2019). Briefly, the spine was oriented 45° above the horizontal to lift the hindlimbs off the ground. A small bandage of adhesive tape was fixed and enveloped around the base of the tail to prevent the risk of injuries. Hindlimb elevation was adjusted to suspend the hindlimb paws while the forelimbs were free to ambulate on the cage. Animal position and welfare were monitored every day and the systems were adjusted if necessary. This protocol has been shown to induce severe muscle deconditioning and skeletal muscle atrophy (Haida et al., 1989).

### 2.4 High‐intensity inclined treadmill running

Following HU, mice in the HU+EX and HU+EX+HT groups underwent 1 week of passive cage reloading, defined as a return to normal cage activity without any experimental intervention. This 1‐week period was chosen to allow safe early recovery after HU, minimizing injury risk while preserving a high level of muscle plasticity prior to subsequent HT and/or exercise (Hanson et al., 2010). After this reloading week, mice in these groups underwent an incremental test on a Monday to assess their supra‐maximal speed (SMS) and personalize their training intensity on an electric treadmill, as previously described (Normand‐Gravier, Solsona, Arnould et al., 2025). After the SMS test, mice performed four additional training sessions every day, from Tuesday to Friday, consisting of high‐intensity inclined treadmill running sessions. Each session included a 5‐min warm‐up at 8 m/min followed by a gradual increase in speed up to SMS and 10 intervals of 20 s at SMS, each followed by 40 s of active recovery at 5 m/min. A 10% reduction of SMS was set to minimize injury risk. The timing, intensity, duration and work to rest ratio of the intervals were chosen to (i) place 20 s bouts in the supramaximal intensity domain, (ii) allow incomplete recovery between bouts to maximize cardiovascular and muscular stress, and (iii) minimize injury risk and overtraining, taking into account HIIT treadmill protocols in mice and general physiological principles derived from interval training studies (Bo et al., 2026; Massett et al., 2021). Treadmill inclination was 25° for both SMS and EX sessions to promote gains in muscle hypertrophy, as previously described (Goh et al., 2019; Seldeen et al., 2018). More specifically, grip strength increased up to 10.9% with 16 weeks of inclined HIIT in aged mice (Seldeen et al., 2018) and functional muscle hypertrophy was induced with 8 weeks of inclined HIIT in adult mice (Goh et al., 2019). Moreover, high‐intensity inclined treadmill running has recently been validated to trigger hypertrophic gains (Murach et al., 2020), as resistance training does. However, EX can also generate responses related to endurance and may be interpreted as a hybrid protocol, due to multiple intervals. In this regard, it has been shown that a 2‐month HIIT training programme performed on an inclined treadmill improves endurance performance in aged mice (Seldeen et al., 2019). Therefore, our EX protocol is believed to promote both resistance and endurance responses, as mentioned by Murach et al. (2020). SMS was not significantly different between HU+EX and HU+EX+HT (36.00 ± 3.34 vs 36.11 ± 2.47 m/min).

### 2.5 HT

Following HU, as for HU+EX and HU+EX+HT, a 1‐week period of normal reloading in a standard cage was performed for HU+HT. After this reloading week, mice in the HU+HT and HU+EX+HT groups were placed in a hot environmental chamber at 40°C for 30 min, 5 times over 1 week, with access to drink and food. In the HU+EX+HT group, HT was performed immediately after EX. This HT protocol was selected to induce a moderate and controlled elevation of core body temperature, sufficient to activate molecular pathways without eliciting overt thermal stress. In rodents, such conditions are known to stimulate heat shock transcription factor 1 activation and HSP expression, which contribute to the maintenance of protein homeostasis and protection against oxidative stress. Repeated short exposures allow sustained activation of these pathways during the early reloading phase following HU, while limiting thermal habituation or excessive systemic strain. This HT protocol was inspired from a previous study that demonstrated beneficial effects on skeletal muscle regeneration, including reduced mitophagy and diminished muscle mass loss during muscle atrophy (Tamura et al., 2015).

### 2.6 Assessment of protein synthesis flux

Protein synthesis flux was measured using the Surface Sensing of Translation (SUnSET) method, as previously described (Schmidt et al., 2009). This method, which utilizes an anti‐puromycin antibody, provides a snapshot of global protein synthesis activity over a short incorporation period immediately prior to tissue sampling and has been validated as an accurate method for measuring protein synthesis flux (Goodman & Hornberger, 2013). Twenty minutes before euthanasia, puromycin (10 mM in phosphate‐buffered saline; PBS) was injected intraperitoneally into the mice (100 µL/25 g).

### 2.7 qPCR analysis

Tissue samples weighing 50 mg were collected for RNA extraction using TRIzol reagent (Thermo Fisher Scientific, Waltham, MA, USA), following the manufacturer's guidelines. RNA concentration and purity (with a 260/280 ratio ≥1.8) were assessed for each sample using a spectrophotometer (BioDrop, Fisher Scientific). Subsequently, 1 µg of total RNA was reverse‐transcribed into complementary DNA (cDNA) utilizing the RevertAid First Strand cDNA Synthesis Kit (Thermo Fisher Scientific) as per the manufacturer's instructions. Primer sequences targeting 5.8S, 18S, 28S rRNA, 45S pre‐rRNA, *UBTF* mRNA, and *B2MG* mRNA were designed using Primer‐BLAST (NCBI, Bethesda, MD, USA), with the following sequences: 5.8S rRNA forward: 5′‐CTTAGCGGTGGATCACTCGG‐3′, reverse: 5′‐ACGCTCAGACAGGCGTAGCC‐3′, 18S rRNA forward: 5′‐AAACGGCTACCACATCCAAG‐3′, reverse: 5′‐GCTGGAATTACCGCGGCT‐3′, 28S rRNA forward: 5′‐GCGGGTGGTAAACTCCATCT‐3′, reverse: 5′‐CACGCCCTCTTGAACTCTCT‐3′, 45S pre‐rRNA forward: 5′‐CTCTTAGATCGATGTGGTGCTC‐3′, reverse: 5′‐GCCCGCTGGCAGAACGAGAAG‐3′, *UBTF* mRNA forward: 5′‐GTTCCAGGGAGAACCCAAG‐3′, reverse: 5′‐TTGACCCAGAGGTCCAAGTG‐3′, *B2MG* mRNA forward: 5′‐CATGGCTCGCTCGGTGAC‐3′, reverse: 5′‐CAGTTCAGTATGTTCGGCTTCC‐3′. Primer specificity was verified by agarose gel electrophoresis, and primer efficiency was determined by calculating the slope of a standard curve generated from serial cDNA dilutions. Primer efficiencies ranged between 80% and 98%. Quantitative reverse transcription–PCR (qRT‐PCR) was conducted using SensiFAST SYBR Hi‐ROX Mix (Bioline, London, UK) on an Applied Biosystems StepOne real‐time PCR system (Thermo Fisher Scientific). The cycling conditions included an initial denaturation at 95°C for 2 min, followed by 40 cycles consisting of 5 s at 95°C for denaturation and 15 s at 60°C for annealing and extension. Samples were processed in clear‐well plates alongside controls for reverse transcription and genomic DNA contamination. Gene expression levels were normalized against the reference gene β2‐microglobulin (*β2MG*), which has been identified as one of the most stable genes in mouse skeletal muscle (Ma et al., 2022). The comparative threshold cycle method ($2^{-\Delta \Delta C_{T}}$) was used to calculate the gene expression (ribosomal RNA content and mRNA expression) of each experimental sample relative to the control samples using the Applied Biosystems StepOne Software (version 2.3).

### 2.8 Western blot analysis

Protein extraction was performed using 30 mg of tissue. Powdered muscle samples were homogenized in a lysis buffer containing 1% Triton X‐100, 20 mM MOPS (pH 7.0), 2 mM EGTA, 5 mM EDTA, 30 mM NaF, 60 mM β‐glycerophosphate, 20 mM sodium pyrophosphate, 1 mM sodium orthovanadate, 1 mM dithiothreitol, and a protease inhibitor cocktail (Roche, Basel, Switzerland; Ref 11836153001). To shear nuclear DNA, homogenates were sonicated on ice four times for 10 s each. After agitating for 1 h, samples were centrifuged at 15,000 *g* for 10 min at 4°C. Protein concentrations in the supernatant were measured using the Bradford assay (Bio‐Rad Laboratories, Hercules, CA, USA, Ref 5000001). Subsequently, 50 µg of protein was mixed with Laemmli sample buffer, separated by SDS–PAGE, and transferred onto nitrocellulose membranes (Amersham, Protran Premium, 0.2 µm; Cytiva, Marlborough, MA, USA). Membranes were stained with Ponceau S before being blocked for 2 h in 5% non‐fat dry milk dissolved in Tris‐buffered saline (TBS)–Tween 0.05% (TBS‐T). Following blocking, membranes were incubated overnight at 4°C with primary antibodies diluted either in 5% non‐fat dry milk or 5% BSA in TBS‐T, according to the manufacturer’s instructions. The antibodies used were against puromycin (dilution 1:2000, cat. no. MABE343, Millipore, USA), ubiquitin (dilution 1:1000, cat. no. z0458, Agilent Dako, Santa Clara, CA, USA), HSP27 (dilution 1:750, cat. no. SPA‐800, Enzo Life Sciences, Farmingdale, NY, USA), HSP70, resulting of gene expression of HSPA1A and HSPA1B (dilution 1:750, cat. no. SPA‐810, Enzo), UBF‐1 (dilution 1:500, cat. no. PA5‐36153, Thermo Fisher Scientific). The following antibodies were purchased from Cell Signaling Technology (Danvers, MA, USA): LC3‐B (dilution 1:1000, cat. no. 2775), SQSTM1/p62 (dilution 1:1000, cat. no. 5114), P‐S6K1 Thr 412 (dilution 1:1000, cat. no. 9206), P‐S6 Ribosomal Protein (P‐RPS6) Ser240/244 (dilution 1:1000, cat. no. 5364), P‐4E‐BP1 Thr37/46 (dilution 1:1000, cat. no. 2855), P‐AMPK Thr172 (dilution 1:750, cat. no. 50081) and P‐DRP1 Ser616 (dilution 1:750, cat. no. 3455). Total and phosphorylated protein levels were normalized based on Ponceau S staining (Sander et al., 2019). Detection was conducted with horseradish peroxidase (HRP)‐conjugated secondary antibodies diluted 1:2000 (Cell Signaling Technology, cat. no. 7074 for rabbit, 7076 for mouse and 7077 for rat). Immunoblots were developed using the Femto West HRP substrate kit (GeneTex, Irvine, CA, USA; Ref GTX14698), and proteins were visualized through enhanced chemiluminescence. Quantification was performed using Image Lab software (Version 5.2.1, Bio‐Rad). The HU+REL group was added after the initial design of the study; therefore, western blot analysis for this group was performed on a separate membrane. However, to allow comparison across membranes, signal intensities were normalized using a common internal control with two samples already loaded. Potential variability in protein loading, transfer efficiency or detection sensitivity across different blots was normalized by using these internal controls. Hence, quantitative comparisons between the HU+REL group and other experimental groups analysed on separate membranes were made possible by normalizing target protein signals to this consistent internal standard. Western blot analyses were conducted using standardized protocols routinely used in the laboratory, with all samples processed in parallel and normalized using appropriate loading controls. All individual animal data were included in the analyses.

### 2.9 Immunoblot analysis of protein carbonyls

Protein carbonylation was evaluated by detecting carbonyl groups using the OxyBlot Protein Oxidation Detection kit (Merck Millipore, Darmstadt, Germany; S7150), following the manufacturer's protocol. Twenty micrograms of solubilized proteins were derivatized with 2,4‐dinitrophenylhydrazine (DNPH) under acidic denaturing conditions. To account for nonspecific antibody interactions, parallel protein samples were acid‐denatured without DNPH treatment. The denatured proteins were separated on an 8% polyacrylamide SDS‐PAGE gel and transferred onto nitrocellulose membranes. Membranes were stained with Ponceau S before being blocked for 1 h in 1% BSA prepared in PBS–Tween (0.05%). Subsequently, blots were incubated overnight at 4°C with the DNPH‐specific primary antibody provided in the kit, diluted in 1% BSA in PBS–Tween. Following washes, membranes were incubated for 1 h at room temperature with the kit's secondary antibody. Detection was conducted using the Femto West HRP substrate kit, and band intensities were quantified with Image Lab software.

### 2.10 Statistical analysis

Statistical analyses were conducted using Jamovi (version 2.3.21) and RStudio (version 2024.12.0). Results are presented as boxplots illustrating the mean, median, quartiles and individual data points. Results are reported as fold‐change ratios, converted as percentage differences. To highlight the effect of suspension, differences between the CTL and HU groups were statistically analysed using Student's *t*‐test (if *P* > 0.05 in the Shapiro–Wilk test for normality and the Levene test for homogeneity of variances) or the Mann–Whitney *U*‐test (if *P *< 0.05 in either the Shapiro–Wilk or Levene test). Then, to assess the effects of the different post‐HU rehabilitation strategies, the HU+HT, HU+EX, HU+EX+HT and HU+REL groups were compared with each other and with the HU group alone (excluding the CTL group). For this purpose, a one‐way ANOVA or a non‐parametric Kruskal–Wallis test was used, depending on data distribution and variance homogeneity. When the *P*‐value was >0.05 for the Shapiro–Wilk and Levene tests, one‐way ANOVA was performed, and Tukey's *post hoc* multiple comparisons tests were used when the *P*‐value was <0.05 for ANOVA F‐test. When conditions were not met for the application of one‐way ANOVA, the non‐parametric Kruskal–Wallis test was performed and the Dwass–Steel–Critchlow–Fligner *post hoc* multiple comparisons test was used when the *P*‐value was below 0.05 for the Kruskal–Wallis test. *P* < 0.05 was considered statistically significant (^*^ *P* < 0.05, ^**^ *P* < 0.01, ^***^ *P* < 0.001). A tendency was considered when *P*‐values were between 0.05 and 0.10 for *post hoc* multiple comparisons tests. When *P*‐values were below 0.10, effect sizes were calculated using Cohen's *d* for ANOVA and Rosenthal's *r* for Kruskal–Wallis tests. The following interpretations of effect size were retained for Cohen's *d*: trivial effect for *d* < 0.10, small effect for 0.10 ≤ *d* < 0.50, medium effect for 0.50 ≤ *d* < 0.80, and large effect for d ≥ 0.80 (Cohen, 1992). For Rosenthal's *r*, the following interpretations of effect size were retained: trivial effect for *r* < 0.10, small effect for 0.10 ≤ *r* < 0.30, medium effect for 0.30 ≤ *r* < 0.50, and large effect for *r* ≥ 0.50 (Cohen, 2013).

## 3 RESULTS

### 3.1 Rectal temperature

Average rectal temperatures were 36.5 ± 0.5°C for CTL, 38.3 ± 0.4°C for HU+EX, 39.7 ± 0.9°C for HU+HT and 39.8 ± 0.9°C for HU+EX+HT. Rectal temperature was higher for HU+HT, HU+EX and HU+EX+HT, compared to CTL (*P* < 0.001, *d* = 4.43 for HU+HT; *P* < 0.001, *d* = 2.46 for HU+EX; and *P* < 0.001, *d* = 4.55 for HU+EX+HT). Moreover, rectal temperature was significantly elevated for HU+HT (*P* = 0.006, *d* = 1.98) and HU+EX+HT (*P* = 0.003, *d* = 2.10), compared to HU+EX.

### 3.2 Effects of HU, HT and EX on markers of cellular stress

**FIGURE 2:**
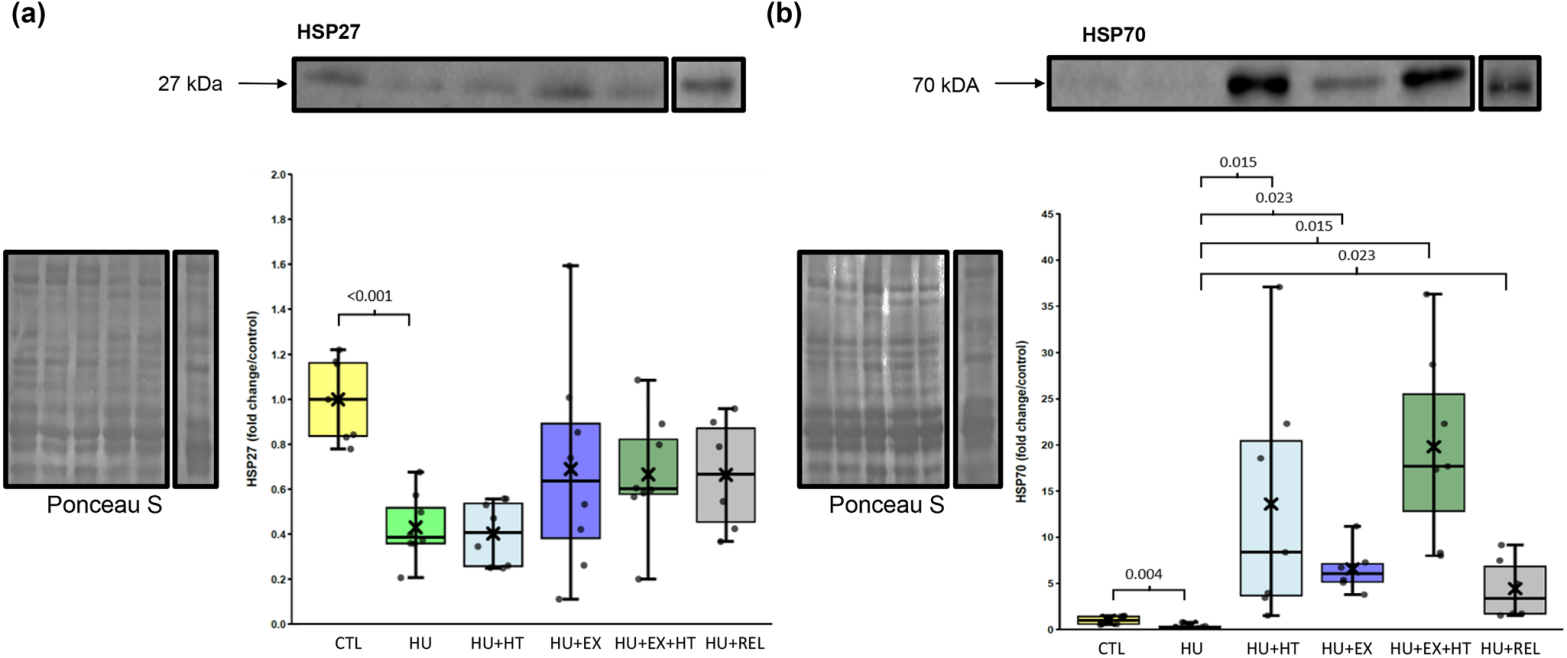
Effects of heat treatment and exercise on heat shock proteins post‐HU. Results are presented as boxplots illustrating the mean, median, quartiles and individual data points. *n* = 6–8 per group. CTL, control; EX, exercise; HT, heat treatment; HU, hindlimb unloading; REL, reloading.

**FIGURE 3:**
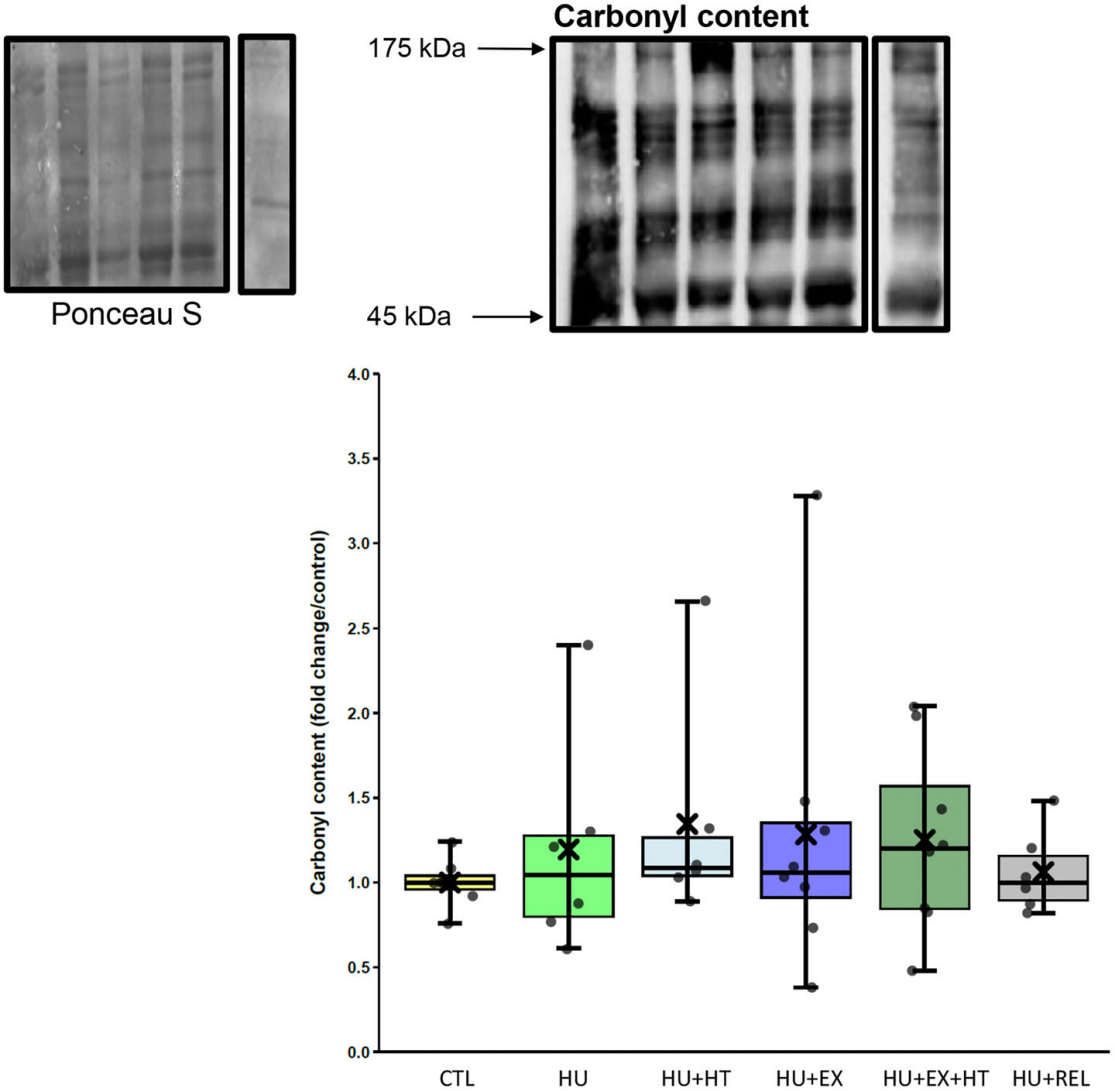
Effects of heat treatment and exercise on carbonylated proteins post‐HU. Results are presented as boxplots illustrating the mean, median, quartiles and individual data points. *n* = 6–8 per group. CTL, control; EX, exercise; HT, heat treatment; HU, hindlimb unloading; REL, reloading.

HSP27 decreased more than 2‐fold for HU, compared to CTL (−57%, *P* < 0.001, *d* = 3.44). No statistically significant difference was found between the HU, HU+REL, HU+HT, HU+EX and HU+EX+HT groups (*P* = 0.072 for Kruskal–Wallis test) (Figure 2a). HSP70 decreased more than 2‐fold for HU, compared to CTL (−65%, *P* = 0.004, *r* = 0.77). Compared to HU, HSP70 increased in HU+REL (+1166%, *P* = 0.023, *r* = 0.90), HU+HT (+3786%, *P* = 0.015, *r* = 0.90), HU+EX (+1777%, *P* = 0.023, *r* = 0.90) and HU+EX+HT (+5560%, *P* = 0.015, *r* = 0.90). Moreover, HSP70 tended to increase in the HU+EX+HT group, compared with HU+REL and HU+EX (*P* = 0.052, *r* = 0.82, compared to both groups) (Figure 2b). Finally, no significant difference was found among the groups in carbonyl content, as determined by DNPH‐based detection (*P* = 0.919) (Figure 3).

### 3.3 Effects of HU, HT and EX on protein synthesis and ubiquitination

**FIGURE 4:**
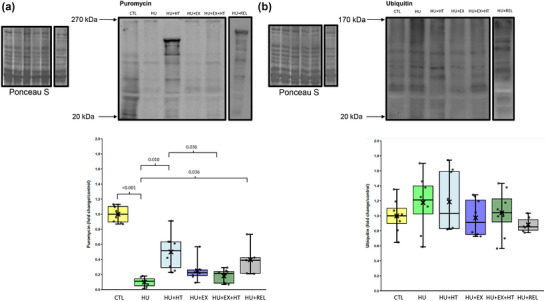
Effects of heat treatment and exercise on protein synthesis and protein ubiquitination post‐HU. Results are presented as boxplots illustrating the mean, median, quartiles and individual data points. *n* = 5–8 per group. CTL, control; EX, exercise; HT, heat treatment; HU, hindlimb unloading; REL, reloading.

Muscle protein synthesis (puromycin incorporation) decreased 10‐fold in HU (−90%, *P* < 0.001, *r* = 0.90), compared to CTL. Compared to HU, muscle protein synthesis increased in HU+HT (+400%, *P* = 0.010, *r* = 0.90), HU+REL (+290%, *P* = 0.036, *r* = 0.90), tended to increase in HU+EX (*P* = 0.069, *r* = 0.74), but did not increase in HU+EX+HT (*P* = 0.376). Finally, muscle protein synthesis increased in HU+HT compared with HU+EX+HT (+178%, *P* = 0.036, *r* = 0.76) and also tended to increase in HU+HT compared with HU+EX (*P* = 0.097, *r* = 0.65) (Figure 4a).

No statistically significant difference was found for ubiquitin labelling (*P* = 0.266) (Figure 4b).

### 3.4 Effects of HU, HT and EX on ribosomal transcription and content

**FIGURE 5:**
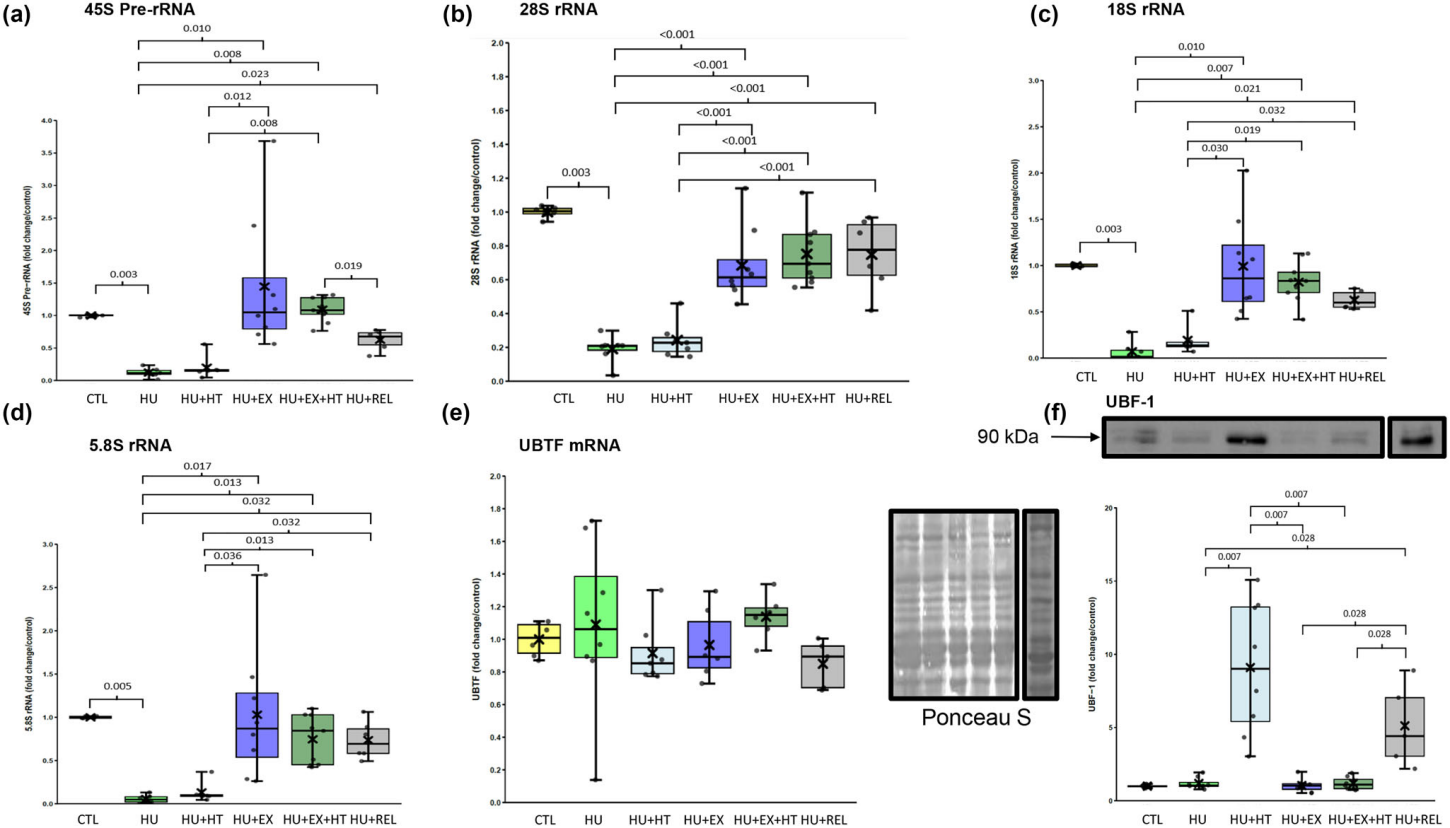
Effects of heat treatment and exercise on ribosomal transcription and content post‐HU. Results are presented as boxplots illustrating the mean, median, quartiles and individual data points. *n* = 5–8 per group. CTL, control; EX, exercise; HT, heat treatment; HU, hindlimb unloading; REL, reloading.

45S pre‐rRNA decreased in HU (−88%, *P* = 0.003, *r* = 0.90), compared with CTL. Compared to HU, 45S pre‐rRNA did not increase in HU+HT (*P* = 0.812) but increased in HU+EX (+1108%, *P* = 0.010, *r* = 0.90), HU+EX+HT (+808%, *P* = 0.008, *r* = 0.89) and HU+REL (+425%, *P* = 0.023, *r* = 0.90). Its expression was also decreased in HU+HT compared with HU+EX (−86%, *P* = 0.012, *r* = 0.90) and tended to decrease in HU+HT compared with HU+REL (*P* = 0.051, *r* = 0.82). Higher expression was also found for HU+EX+HT concerning 45S pre‐rRNA, compared with HU+HT (+445%, *P* = 0.008, *r* = 0.89) and HU+REL (+73%, *P* = 0.019, *r* = 0.85) (Figure 5a). 28S rRNA content was decreased in HU (−81%, *P* = 0.003, *r* = 0.90), compared with CTL. Compared to HU, 28S rRNA increased in HU+EX (+263%, *P* < 0.001, *d* = 2.87), HU+EX+HT (+295%, *P* < 0.001, *d* = 3.27) and HU+REL (+295%, *P* < 0.001, *d* = 3.25) but did not increase in HU+HT (*P* = 0.978). Finally, 28S rRNA decreased in HU+HT, compared with HU+EX (−65%, *P* < 0.001, *d* = 2.56), HU+EX+HT (−68%, *P* < 0.001, *d* = 2.96) and HU+REL (−68%, *P* < 0.001, *d* = 2.94) (Figure 5b). 18S rRNA decreased in HU (−93%, *P* = 0.003, *r* = 0.90), compared with CTL. Moreover, compared with HU, 18S rRNA increased in HU+EX (+1314%, *P* = 0.010, *r* = 0.90), HU+EX+HT (+1071%, *P* = 0.007, *r* = 0.89) and HU+REL (+800%, *P* = 0.021, *r* = 0.90), but did not increase in HU+HT (*P* = 0.226). Finally, 18S rRNA level was lower in HU+HT, compared with HU+EX (−81%, *P* = 0.030, *r* = 0.82), HU+EX+HT (−77%, *P* = 0.019, *r* = 0.85) and HU+REL (−70%, *P* = 0.032, *r* = 0.91) (Figure 5c). 5.8S rRNA decreased in HU (−94%, *P* = 0.005, *r* = 0.91), compared with CTL. Compared with HU, 5.8S rRNA content increased in HU+EX (+1617%, *P* = 0.017, *r* = 0.89), HU+EX+HT (+1150%, *P* = 0.013, *r* = 0.88) and HU+REL (+1133%, *P* = 0.032, *r* = 0.91), but did not increase in HU+HT (*P* = 0.444). Compared with HU+HT, higher expression was found for HU+EX (+692%, *P* = 0.036, *r* = 0.82), HU+EX+HT (+477%, *P* = 0.013, *r* = 0.88) and HU+REL (+469%, *P* = 0.032, *r* = 0.91) concerning 5.8S rRNA (Figure 5d). No statistically significant difference was found between HU and CTL (*P* = 0.430) for UBF‐1 protein expression. Compared with HU, UBF‐1 increased in HU+HT (+665%, *P* = 0.007, *r* = 0.90) and HU+REL (+330%, *P* = 0.028, *r* = 0.88), but did not increase in HU+EX (*P* = 0.996) and HU+EX+HT (*P* = 1.000). Compared with HU+HT, lower expression was found for UBF‐1 in HU+EX (−89%, *P* = 0.007, *r* = 0.90) and HU+EX+HT (−87%, *P* = 0.007, *r* = 0.90). Moreover, higher expression was found for UBF‐1 protein in HU+REL, compared with HU+EX (+392%, *P* = 0.028, *r* = 0.88) and HU+EX+HT (+327%, *P* = 0.028, *r* = 0.88) (Figure 5f). Finally, no statistically significant difference was found for *UBTF* mRNA expression (*P* = 0.130) (Figure 5e).

### 3.5 Effects of HU, HT and EX on the mTOR pathway

**FIGURE 6:**
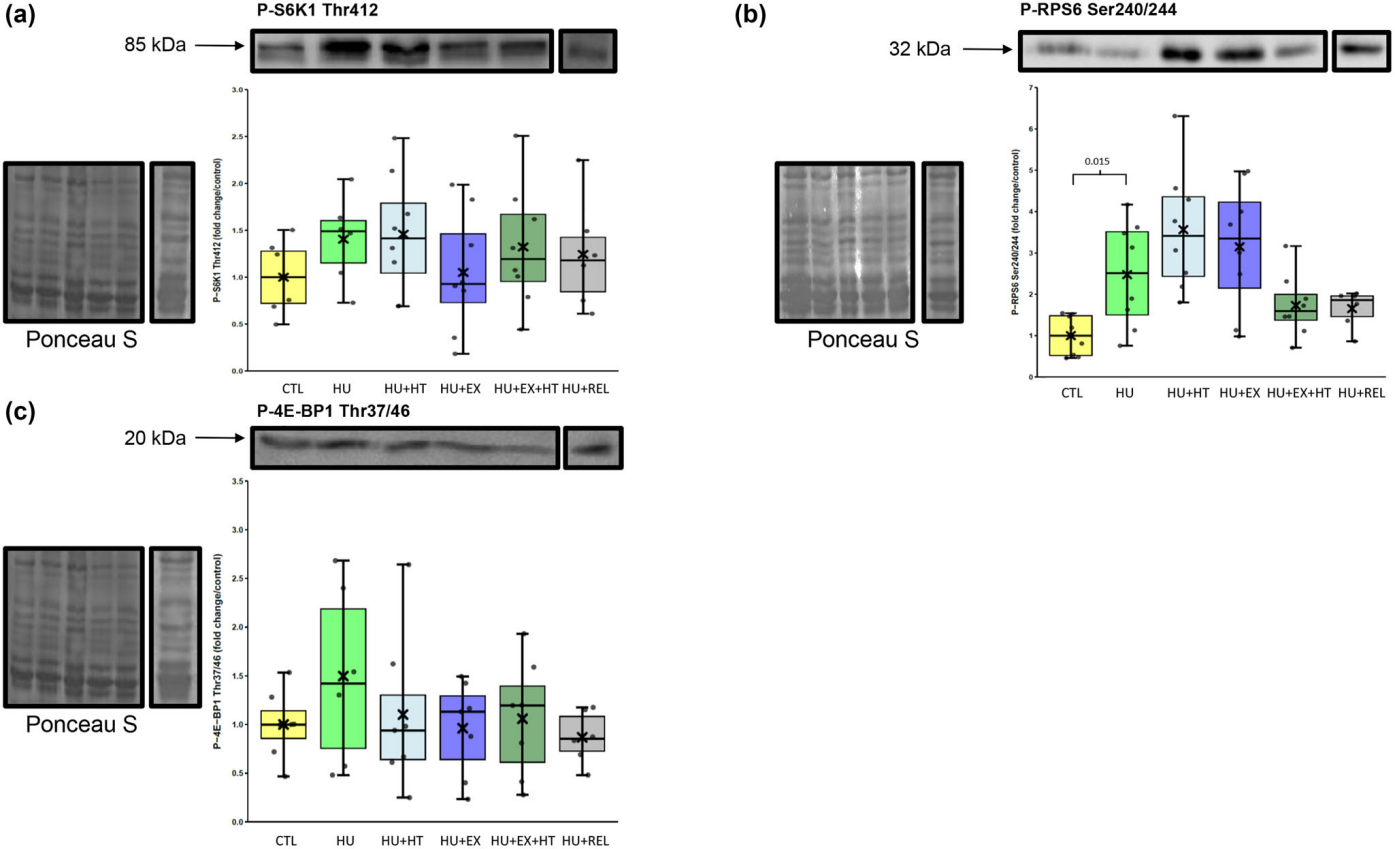
Effects of heat treatment and exercise on mTORC1 pathway post‐HU. Results are presented as boxplots illustrating the mean, median, quartiles and individual data points. *n* = 5–8 per group. CTL, control; EX, exercise; HT, heat treatment; HU, hindlimb unloading; REL, reloading.

No statistically significant difference was observed concerning P‐S6K1 Thr412 (*P* = 0.104) (Figure 6a). P‐RPS6 Ser240/244 increased in the HU group (+148%, *P* = 0.015, *r* = 0.65), compared to CTL. Moreover, P‐RPS6 Ser240/244 tended to increase in HU+HT, compared with HU+REL (*P* = 0.052, *r* = 0.78) and HU+EX+HT (*P* = 0.065, *r* = 0.70) (Figure 6b). No statistically significant difference was observed concerning P‐4EBP1 Thr37/46 (*P* = 0.281) (Figure 6c).

### 3.6 Effects of HU, HT and EX on autophagy and mitochondrial fission markers

**FIGURE 7:**
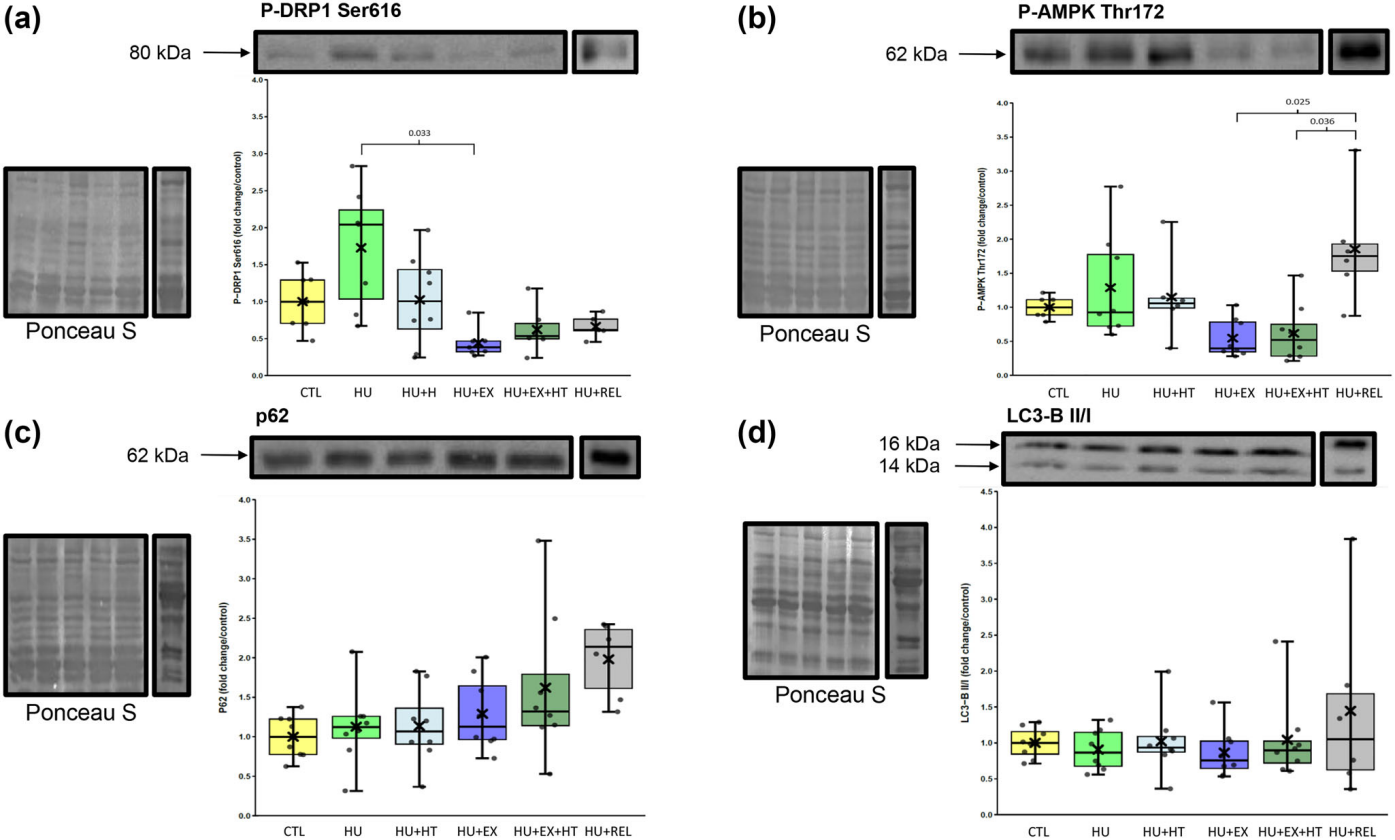
Effects of heat treatment and exercise on autophagy and mitochondrial fission markers post‐HU. Results are presented as boxplots illustrating the mean, median, quartiles and individual data points. *n* = 5–8 per group. CTL, control; EX, exercise; HT, heat treatment; HU, hindlimb unloading; REL, reloading.

No statistical significance was found between HU and CTL for P‐DRP1 Ser616 (*P* = 0.165). Compared to HU, P‐DRP1 Ser616 decreased in HU+EX (−75%, *P* = 0.033, *r* = 0.83) and tended to decrease in HU+EX+HT (*P* = 0.076, *r* = 0.78), but did not decrease in HU+HT (*P* = 0.447) and HU+REL (*P* = 0.128) (Figure 7a). No statistical significance was found between HU and CTL for P‐AMPK Thr172 (*P* = 0.954). Compared to HU+REL, P‐AMPK Thr172 decreased in the HU+EX group (−70%, *P* = 0.025, *r* = 0.86) and in the HU+EX+HT group (−67%, *P* = 0.036, r = 0.82) (Figure 7b). No statistically significant difference was found between HU and CTL for p62 (*P* = 0.533). Furthermore, p62 protein expression was not statistically different between HU, HU+HT, HU+EX, HU+EX+HT and HU+REL (*P* = 0.061 for ANOVA F‐test) (Figure 7c). Finally, no statistical significance was found between groups for LC3‐B II/I (*P* = 0.478) (Figure 7d).

## 4 DISCUSSION

The study aimed to examine the impact of a 3‐week period of HU and subsequent reloading strategies (i.e., HT, hybrid exercise and a sequential application of both stressors) on markers of protein turnover. Our results indicate that HU decreased global protein synthesis, rRNA content and content of chaperone proteins HSP27 and HSP70. Furthermore, whereas HT and EX applied individually induced only a few additional effects compared to normal reloading, the use of both stressors seemed to be deleterious, as evidenced through the absence of an increase in protein synthesis flux.

### 4.1 HU decreases protein synthesis, rRNA content and HSPs

It has been previously reported that HU leads to decreased strength, muscle mass, CSA and protein synthesis along with increased protein degradation (Baehr et al., 2017; Haida et al., 1989; Oliveira et al., 2019). We also observed a decrease in puromycin incorporation, which is in concordance with previous findings, where protein synthesis rates were decreased after HU or immobilization in mice (Baehr et al., 2016, 2017; You et al., 2015). Seven days of HU have been shown to reduce total RNA concentration and 47S pre‐rRNA, leading to impaired translation capacity and promotion of muscle atrophy (Figueir et al., 2021). In the current study, we also observed a decrease in 5.8S, 18S and 28S rRNA and 45S pre‐rRNA expression with HU. Furthermore, our results reported that HU led to a decrease in HSP27 and HSP70 protein levels, which is consistent with previous findings (Lawler et al., 2006; Naito et al., 2000). The observed decrease in HSPs may further exacerbate skeletal muscle atrophy. Hence, HU results in a significant reduction in total rRNA content, diminishing translational capacity and subsequent protein synthesis flux, as observed with puromycin incorporation. Taken together, the observed reductions in protein synthesis flux, rRNA content and HSP levels explain the skeletal muscle atrophy induced by HU.

### 4.2 Impact of HT on protein synthesis, rRNA content and HSPs

Previous studies have shown that HT can attenuate the loss of muscle CSA and muscle mass during periods of immobilization, as demonstrated in both animal and human research (Hafen et al., 2019; Labidi et al., 2024; Selsby & Dodd, 2005). We observed an increase in muscle protein synthesis in the HU+HT group compared with the HU group. However, muscle protein synthesis was also increased in the HU+REL group compared with the HU group. These results indicate that, at this specific recovery time point (14 days post‐HU), repeated heat exposure did not produce additional effects compared with passive reloading (HU+REL) on protein synthesis rates. It has previously been shown that the increase in protein synthesis is strongly correlated with elevated P‐RPS6 levels (Stewart & Thomas, 1994). In the present study, HT applied after HU tended to increase P‐RPS6 compared with the HU+REL group. Previous work has also reported that heat stress (41°C for 60 mins) increases HSP70 expression after 5 days of HU, indicating enhanced protein synthesis (Goto et al., 2004). Moreover, HSP70 overexpression has been shown to promote recovery of muscle fibre CSA following immobilization in mice (Miyabara et al., 2012). In our study, HSP70 protein levels increased markedly with HT compared with HU, but also increased in the HU+REL group compared with HU. The combined increase in P‐RPS6 and HSP70 protein expression observed only in the HU+HT group may therefore indicate a strong anabolic stimulus induced by post‐HU HT. However, passive cage recovery alone was sufficient to increase protein synthesis rates following HU, making it difficult to conclude that repeated post‐HU heat exposure provided any additional benefit to protein synthesis. While we might have expected, as observed in the HU condition, that muscle protein synthesis would closely correlate with total RNA content (Millward et al., 1973), we did not observe any increase in 5.8S rRNA, 18S rRNA, 28S rRNA or pre‐rRNA 45S in the HU+HT group compared with HU. This discrepancy between rRNA content and protein synthesis may be attributed to post‐transcriptional or translational regulation (Sonenberg & Hinnebusch, 2009), explaining why protein synthesis rates can increase without a corresponding rise in rRNA content. In this context, reduced rRNA levels could be compensated by enhanced ribosomal activity, leading to an increased rate of protein translation (Rodnina, 2016). A recent study (Kotani et al., 2024) reported that, unlike resistance exercise, neither isolated HWI nor HWI applied post‐exercise increased rRNA or pre‐rRNA 45S expression. In fact, 18S and 28S rRNA levels tended to decrease in the HWI group (Kotani et al., 2024). The authors suggested that the cellular mechanisms underlying heat‐induced hypertrophy differ from those associated with resistance exercise and may involve a reduction in ribophagy. This could explain why protein synthesis rates increase without a corresponding rise in rRNA content, through enhanced ribosomal functional stability. In summary, this study demonstrates for the first time that the increase in muscle protein synthesis is not necessarily associated with elevated rRNA content 14 days post‐HU, and that HT exerts limited additional effects compared with REL at this stage of recovery (14 days post‐HU). Indeed, protein synthesis rates were similarly increased in the HU+HT and HU+REL groups, although only HT induced a concomitant increase in HSP70 and P‐RPS6.

### 4.3 Impact of EX on protein synthesis, rRNA content and HSPs

In our study, HU+EX did not induce any increase in the protein expression of protein synthesis markers (mTORC1 pathway) or in protein synthesis rates (puromycin incorporation) compared with the HU group, although a trend toward an increase was observed for puromycin. Treadmill running was chosen to individualize the training load and minimize interindividual variability in the distance covered, which is often observed with voluntary wheel running (Patel et al., 2019). However, it has been reported that treadmill running after a period of HU can induce muscle damage, potentially impairing muscle mass recovery in this type of protocol (Kasper et al., 1990). Although the training load was individualized and adjusted by reducing the supramaximal speed (SMS) by 10% to prevent overtraining and injury, we implemented a high‐intensity training protocol, which may have generated excessive stress on an already atrophied gastrocnemius muscle. This unexpected result could also be explained by an acute inflammatory response (i.e., the release of pro‐inflammatory cytokines such as IL‐1β or IL‐6) induced by high‐intensity exercise (Cerqueira et al., 2019), combined with insufficient recovery time between sessions, leading to excessive cellular stress and an attenuated effect of exercise on protein synthesis post‐HU. However, the timing of sampling (14 days after HU) could also account for this response. Indeed, it has been shown that after 28 days of HU, daily treadmill running for 14 days led to a decrease in muscle fibre CSA compared to the sedentary group, whereas muscle mass significantly increased in rats that ran daily for 28 days post‐HU (Kasper et al., 1990). Finally, the current exercise protocol represents a hybrid retraining strategy promoting both endurance and strength‐type adaptations, which may result in a more modest increase in muscle protein synthesis compared to resistance training alone. It was recently shown that resistance training can increase total RNA content as well as 28S rRNA (Hammarström et al., 2022). Moreover, 18S and 28S rRNA levels remained elevated after 8 days of rest following resistance training, and total RNA content was strongly correlated with training‐induced hypertrophy (Hammarström et al., 2022). We also found that post‐HU exercise increased the levels of 5.8S, 18S and 28S rRNAs and pre‐rRNA 45S compared to the HU group. However, passive reloading (HU+REL) also induced an increase in these markers. Consistent with these observations, previous work reported that after 7 days of HU, a reloading period of the same duration increased pre‐rRNA 47S content compared with HU (Figueir et al., 2021). Thus, passive reloading alone is sufficient to increase rRNA content, and our hybrid exercise protocol did not induce additional effects on these markers. In our study, as also observed in the HU+HT group (but in the opposite direction), the increase in rRNA content was not correlated with an increase in muscle protein synthesis. We also found that HSP70 protein expression was elevated in the HU+EX group compared to HU, but it was similarly increased in the HU+REL group. This result is consistent with a study showing a similar increase in HSP70 protein expression in mice subjected to uphill treadmill running (twice daily for 6 days) compared with those undergoing passive reloading after 28 days of immobilization in rats (Venojärvi et al., 2007). Thus, these results show that hybrid retraining is not superior to passive reloading (HU+REL) in terms of protein synthesis rates, rRNA content or HSP protein expression at this specific time point post‐HU. It has recently been shown that overexpression of DRP1, a key regulator of mitochondrial fission, can promote muscle atrophy by disrupting mitochondrial dynamics (Dulac et al., 2021), although its deletion also promotes muscle atrophy (Dulac et al., 2020). Conversely, a moderate decrease in its phosphorylation appears to be beneficial. Indeed, endurance training has been shown to decrease DRP1 phosphorylation, consistent with reduced mitochondrial fission and potentially improved muscle function (Fealy et al., 2014). Moreover, increased AMPK activity promotes mitochondrial fission (Crosas‐Molist et al., 2023), whereas reduced fission can be associated with decreased AMPK activity. In our study, we observed a decrease in P‐DRP1 with post‐HU exercise compared to HU. A decrease in P‐AMPK was also observed in the HU+EX group compared to HU+REL. This concomitant decrease in P‐DRP1 and P‐AMPK observed in the HU+EX group suggests that hybrid retraining may reduce mitochondrial fission after HU, potentially improving muscle function through more sustainable and efficient mitochondria (Fealy et al., 2014). In summary, our results demonstrate that hybrid retraining after HU does not induce additional effects compared to passive reloading in terms of protein synthesis rates, rRNA content or HSP expression at this specific sampling time. However, HU+EX reduced DRP1 and AMPK phosphorylation, indicating decreased mitochondrial fission and improved mitochondrial efficiency following hybrid retraining.

### 4.4 Impact of EX+HT on protein synthesis, rRNA content and HSPs

Regarding the results for HU+EX+HT, our study revealed that the sequential use of the two stressors did not increase muscle protein synthesis compared to the HU group. A previous study showed that combining HT with high‐intensity exercise did not restore the muscle mass‐to‐body weight ratio or the diameters of type IIa and IIb muscle fibres, whereas the combination of HT and low‐intensity exercise partially restored these parameters in rats (Yoshida et al., 2013). The authors of that study proposed that combining HT with high‐intensity exercise may not be optimal for muscle mass recovery following atrophy (Yoshida et al., 2013). In line with these findings, our data indicate that post‐exercise HT does not provide additional benefits after HU and may even have a potentially detrimental effect when high‐intensity exercise is applied following HU. In this regard, protein synthesis fluxes were reduced in the HU+EX+HT condition compared to HU+HT, as was RPS6 phosphorylation, which also tended to decrease compared to HU+HT. This suggests that the application of the two stressors may even inhibit the positive effects of HT or REL on muscle anabolism. Thus, heat exposure alone appears to induce few additional effects compared to the REL group but is not detrimental in the absence of activity, whereas it may become harmful when applied immediately after high‐intensity exercise in the context of disuse‐induced atrophy. In this sense, as proposed by Labidi and colleagues, there may be a dose–response relationship regarding the effects of heat: low doses of heat may be beneficial in the absence of activity, while higher doses may be required in the presence of activity, and no further benefit from heat is observed when a stimulus such as exercise is applied (Labidi et al., 2021). This potential dose–response relationship could therefore explain why, in a context of muscle atrophy (and thus inactivity), post‐exercise heat exposure appears to be detrimental, generating excessive cellular stress in atrophied muscle. While exercise markedly increases AMPK activity through its phosphorylation at Thr172 (Spaulding & Yan, 2022), heat stress strongly enhances HSP70 expression (Goto et al., 2004). Consistent with this, in our study, applying heat after exercise induced a strong increase in HSP70 protein expression compared to the HU group, and it also tended to increase compared to the HU+REL and HU+EX groups, promoting an additive effect of the two stressors on HSP70 content. We may therefore hypothesize that sequentially applying the two stressors generates signalling interference between these pathways, leading to an adaptive conflict. Indeed, overexpression of HSP70 has been shown to inhibit AMPK‐mediated autophagy (Alhasan et al., 2024). However, autophagy is essential for skeletal muscle plasticity in response to exercise and supports the physiological adaptations induced by endurance training (Sanchez et al., 2014). Thus, a reduction in this pathway's activity could lead to the accumulation of dysfunctional organelles within muscle cells, thereby limiting cellular adaptations via AMPK‐dependent modulation of autophagy. Consequently, the marked increase in HSP70 (+5560%) observed in HU+EX+HT may have been excessive and may have inhibited autophagy through AMPK. However, p62 and LC3‐B II/I levels remained unchanged, suggesting that the alteration of the autophagic flux occurred at an early stage of recovery, following each EX+HT session (within the first hours post‐exercise + heat), thereby limiting acute organelle remodelling via HSP70–AMPK interference. The repeated impairment of autophagic flux after each exercise + heat stimulus could then contribute to the lack of increase in protein synthesis observed in the HU+EX+HT group, due to the accumulation of dysfunctional organelles such as ribosomes. Moreover, a recent study showed that the use of hybrid exercise and heat exposure increased a marker of autophagic flux and cellular stress (elevated LC3‐B II/I ratio, increased NF‐κB phosphorylation at Ser536, and a trend toward increased DRP1 phosphorylation at Ser616) in the quadriceps 4 h post EX+HT (Normand‐Gravier, Solsona, Arnould et al., 2025). Thus, another possible explanation is that applying the two stressors induced excessive cellular stress and an overly elevated autophagic flux after each session, leading to excessive degradation of muscle organelles and, ultimately, impaired protein synthesis. Nevertheless, 5.8S, 18S and 28S rRNA contents were also elevated in this condition compared with HU, although this increase was not significantly greater than that observed in HU+EX and HU+REL. In contrast, pre‐rRNA 45S content, a marker of ribosomal transcription, was significantly increased in the HU+EX+HT group compared to HU and also compared to HU+REL. This novel finding indicates that post‐exercise heat exposure after HU could enhance ribosomal transcription to a greater extent than exercise alone, potentially improving the muscle cell's translational capacity in the long term. However, protein synthesis rates were not significantly increased in this condition, suggesting that the rise in pre‐rRNA 45S content might represent a compensatory mechanism to counterbalance the impaired protein synthesis observed at this specific time point (14 days post‐HU). Finally, the effect of exercise alone on mitochondrial fission was also observed in the HU+EX+HT condition, with a trend toward decreased P‐DRP1 compared to HU and reduced P‐AMPK compared to HU+REL, but no additional effects were found. In summary, these results indicate that, following a period of HU, post‐exercise heat application does not induce additional effects on rRNA content, HSPs or mitochondrial markers compared to the group that performed hybrid retraining alone, although a greater increase in ribosomal transcription (pre‐rRNA 45S) was observed when using the two stressors. However, at this sampling time point, HU+EX+HT appears to inhibit the increase in protein synthesis induced by heat therapy alone or by passive reloading.

### 4.5 Protein degradation markers and oxidative stress are not affected by unloading, HT or exercise

Protein degradation is partially regulated by the ubiquitin–proteasome system. An increased activation of this system promotes muscle atrophy through an increased degradation of ubiquitinated proteins (Pang et al., 2023). Conversely, in our study, we did not observe an increase in total ubiquitinated protein levels with HU or different reloading strategies. This result is consistent with another study where total ubiquitinated protein levels were not increased in soleus after 14 days of HU (Baehr et al., 2016). However, we cannot conclude firmly that HU and post‐HU EX and HT have no impact on the ubiquitin–proteasome system, as a study found that the two key atrophic markers, E3 ubiquitin ligases muscle RING finger 1 (MuRF1) and muscle atrophy F‐box (MAFbx), were increased during immobilization (Bodine & Baehr, 2014).

Autophagy represents another important cellular degradation system by removing damaged cellular components (Sanchez et al., 2014). However, in our study none of the experimental conditions affected the expression of the two key autophagic markers LC3‐B II/I and p62. In this sense, a review stated that muscle atrophy induced by prolonged HU (>14 days) is more related to a decrease in protein synthesis than an increase in protein degradation (Baehr et al., 2017). Taken together, these data suggest that muscle mass modulation during prolonged suspension and reloading is more likely related to variations in muscle protein synthesis.

Concerning global oxidative stress, measured across protein carbonylation, no modification has been observed under any experimental conditions. A study found that oxidative stress, measured through superoxide dismutase and catalase activities, was increased after 5 days of reloading following 14 days of HU (Andrianjafiniony et al., 2010). However, the activity of these oxidative stress markers returned to baseline values after 14 days of reloading (Andrianjafiniony et al., 2010). Hence, our HU and reloading durations (3 weeks and 2 weeks, respectively) could have missed the peak expression window of oxidative stress markers. Moreover, these findings suggest that HU and the different reloading strategies used in our study (HT or EX) are not sufficient to increase overall oxidative stress, even though specific oxidative stress markers could be significantly modified during the early stages of HU or reloading.

### 4.6 Limitations and perspectives

There are some limitations to this study. First, although we showed that HT and EX are promising reloading strategies, normal reloading also contributes to the observed recovery. Indeed, muscle protein synthesis increased in the HU+REL group and rRNA content was restored. Previous studies have shown that passive reloading can increase protein synthesis or ribosome biogenesis following HU (Baehr et al., 2016, 2017; Figueir et al., 2021). Hence, the effects observed in the HU+HT and HU+EX groups could be related to the combined effects of passive reloading and HT or EX. It is also important to note that substantial inter‐individual variability exists even in isogenic mouse models. Epigenetic differences, variability in stress response, social interactions within the cage and other environmental factors can contribute to differences in gene expression among genetically identical animals (Krishnan et al., 2007; Loos et al., 2015; Rakyan et al., 2002). We hypothesize that earlier sampling (e.g., 7 days post‐HU) could have revealed an additional effect of heat compared to the HU+REL group. It has also been shown that HWI provides higher thermal conductivity than air, inducing a greater increase in body temperature compared with a sauna (Atencio et al., 2025), suggesting that localized HWI could have induced more effects than a whole‐body heat chamber. Results should also be interpreted cautiously given the relatively small sample size for each group (*n* = 8 or *n* = 6) and the work can be considered as a pilot study. Further studies with larger sample size are required to extend and consolidate our results. Moreover, further studies should include functional assessments, such as grip strength, fatigue resistance or CSA, to draw firm conclusions on the impact of HT and EX on muscle recovery. Future studies should also precisely examine the impact of sex on muscle recovery following HU. Surprisingly, while HT did not restore rRNA content, it increased UBF‐1 protein expression more than 9‐fold, with a concomitant increase in muscle protein synthesis and HSP levels. As previously reported, cellular responses explaining heat‐induced hypertrophy are different from those observed with exercise and can be linked to a decreased ribophagy (Figueiredo & McCarthy, 2019). Moreover, as HSPs prevent the loss of function of other proteins (Dahiya & Buchner, 2019), future studies should examine the potential link between HSPs and ribosome turnover under heat exposure, potentially explaining cellular and physiological responses observed with regular heating (i.e., increase in muscle protein synthesis and CSA).

### 4.7 Conclusions

This study shows that, in mice, post‐HU heat therapy (HU+HT) does not induce additional effects compared with the group undergoing a normal reloading (HU+REL). Increases in protein synthesis flux were similar between these two groups, although one protein synthesis marker (P‐RPS6) tended to increase in the HU+HT group compared with HU+REL. Moreover, heat therapy did not increase rRNA content. Hybrid retraining, for its part, led to a simultaneous decrease in the protein expression of the mitochondrial fission marker P‐DRP1 and of P‐AMPK, suggesting a reduction in mitochondrial fission. However, no additional effects were found regarding rRNA and HSPs compared with the HU+REL group. Finally, the sequential application of exercise and heat post‐HU appears to be detrimental, since protein synthesis flux did not increase in this condition, even though rRNA content increased in this group. Thus, at this sampling time point (14 days post‐HU), heat therapy and hybrid retraining produce only minor additional effects on the markers studied, compared with normal reloading (REL). In contrast, sequentially applying the two stresses may be detrimental, as evidenced by the absence of an increase in protein synthesis flux. Therefore, determining the optimal dose and timing of sequential exercise and heat exposure is crucial for maximizing muscle recovery following periods of unloading.

## AUTHOR CONTRIBUTIONS

Conception or design of the work: Tom Normand‐Gravier, Robert Solsona, Henri Bernardi, Anthony M. J. Sanchez. Acquisition or analysis or interpretation of data for the work: Tom Normand‐Gravier, Robert Solsona, Christelle Bertrand‐Gaday, Margot Issertine, Florence Sabatier, Flavie Arnould, Sébastien Racinais, Fabio Borrani, Henri Bernardi, Anthony M. J. Sanchez. Drafting the work or revising it critically for important intellectual content: Tom Normand‐Gravier, Robert Solsona, Christelle Bertrand‐Gaday, Margot Issertine, Florence Sabatier, Flavie Arnould, Sébastien Racinais, Fabio Borrani, Henri Bernardi, Anthony M. J. Sanchez. Final approval of the version to be published: Tom Normand‐Gravier, Robert Solsona, Christelle Bertrand‐Gaday, Margot Issertine, Florence Sabatier, Flavie Arnould, Sébastien Racinais, Fabio Borrani, Henri Bernardi, Anthony M. J. Sanchez. Agreement to be accountable for all aspects of the work: Tom Normand‐Gravier, Robert Solsona, Christelle Bertrand‐Gaday, Margot Issertine, Florence Sabatier, Flavie Arnould, Sébastien Racinais, Fabio Borrani, Henri Bernardi, Anthony M. J. Sanchez. All the authors approved the final version of the manuscript and agree to be accountable for all aspects of the work in ensuring that questions related to the accuracy or integrity of any part of the work are appropriately investigated and resolved. All the persons designated as authors qualify for authorship, and all those who qualify for authorship are listed.

## CONFLICT OF INTEREST

The authors confirm that this article has no financial or non‐financial interests that are directly or indirectly related to the work submitted for publication.

## Data Availability

The datasets used and/or analysed during the present study will be publicly available online (link for downloading all original data from the study (qPCR and Western Blot) after the acceptance of the manuscript).
